# Factors associated with parenting self‐efficacy: A systematic review

**DOI:** 10.1111/jan.14767

**Published:** 2021-02-15

**Authors:** Yuan Fang, Mirte Boelens, Dafna A. Windhorst, Hein Raat, Amy van Grieken

**Affiliations:** ^1^ Department of Public Health Erasmus MC University Medical Center Rotterdam The Netherlands

**Keywords:** nursing, parenting, process model, self‐efficacy, systematic review

## Abstract

**Aims:**

To provide an overview of the parental, child, and socio‐contextual factors related to general parenting self‐efficacy (PSE) in the general population.

**Design:**

Systematic review.

**Data sources:**

Medline Ovid, Web of Science, Embase, and PsycINFO Ovid were systematically searched for studies published between January 1980‐June 2020.

**Review Methods:**

Studies were included if they described associations between factor(s) and PSE among parents of children aged 0–18 years old in the general population, and published in an English language peer‐reviewed journal. Studies with participants from specific populations, studies describing the development of instruments for PSE, qualitative studies, reviews, theses, conference papers and book chapters were excluded. Belsky's process model of parenting guided the data synthesis.

**Results:**

Of 3,819 articles, 30 articles met the inclusion criteria. Eighty‐nine factors were identified. There was evidence of associations between child temperament, maternal parenting satisfaction, parenting stress, maternal depression, household income, perceived social support and PSE. Evidence was inconsistent for an association of educational level, parity, number of children in the household and PSE in mothers. There was no evidence of an association for child gender, age, marital status and PSE in both mothers and fathers; ethnicity, age, employment status in mothers; household income in fathers; and educational level, parenting fatigue in parents.

**Conclusion:**

A range of factors studied in relation to PSE was identified in this systematic review. However, the majority of the factors was reported by one or two studies often implementing a cross‐sectional design.

**Impact:**

There is some evidence for an association between some potentially modifiable factors and PSE in the general population, this information may be used by health and social professionals supporting child health and well‐being. Future longitudinal studies are recommended to study parental, child and socio‐contextual factors associated with PSE to inform the development of intervention strategies.

## 1 INTRODUCTION

Parenting self‐efficacy (PSE) underlies parents’ confidence to raise their child (Bandura, 1977; Montigny & Lacharite, 2005). The existing literature has highlighted associations between PSE and health outcomes in both parents and children (Albanese et al., 2019; Jones & Prinz, 2005; Sanders & Woolley, 2005). High PSE has been related to less depression, anxiety, stress in parents and fewer behaviour problems, better overall development in children (Albanese et al., 2019; Jones & Prinz, 2005). Conversely, low PSE is considered to be a risk factor of negative parenting and a negative parent–child relationship (Albanese et al., 2019). Apart from direct effects on parenting, PSE has also been shown to mediate the effects that parental depression and child temperament can have on parenting (Teti & Gelfand, 1991), and to buffer the impact of adversity brought on by an undesirable living environment (e.g. adverse housing conditions) (Ardelt & Eccles, 2001). Therefore, identifying factors associated with PSE can be important for youth health care professionals, as well as for the development and tailoring of interventions aiming to support parents.

## 2 BACKGROUND

As a subcategory of general self‐efficacy, PSE has been defined as beliefs or judgements a parent holds regarding their capabilities to organize and execute a set of tasks related to parenting a child (Bandura, 1977; Montigny & Lacharite, 2005). Three levels of PSE have been distinguished in previous literature: general, narrow domain and task‐specific (Coleman & Karraker, 2000, 2003). General PSE refers to parents' perceptions of their ability to engage in the behaviours expected in their role as parents without focusing on specific tasks, i.e., general parenting situations across child ages (Jones & Prinz, 2005). Narrow‐domain PSE concentrates on parental perceived competence in one parenting domain, such as involvement in school‐related activities. Finally, task‐specific PSE refers to the confidence a parent has over a set of discrete parenting tasks, for example, breastfeeding and soothing a baby. In the current review, we study the general level PSE. This level of PSE is considered a less sensitive measure to assess changes in PSE compared with task‐specific level PSE (Bandura, 1977), however, it is applicable for a broader range of studies with a broader range of child ages (Baker et al., 2013; Črnčec et al., 2010; Cutrona & Troutman, 1986; de Haan et al., 2013; Murdock, 2013; Teti & Gelfand, 1991; Troutman et al., 2012).

Previous studies have identified a broad range of factors associated with general PSE, including parenting psychological well‐being (e.g., stress, depression) (Dunning & Giallo, 2012; Forehand et al., 2012; Gordo et al., 2018; Jover et al., 2014; Slomian et al., 2019), social support, marital quality, child temperament and child behavioural difficulties (Cutrona & Troutman, 1986; de Haan et al., 2013; Murdock, 2013; Teti & Gelfand, 1991). Other factors under study, including general health status, household income, socioeconomic status, birth weight, gestational weeks and parity, have thus far been inconsistently associated with PSE (Baker et al., 2013; Cutrona & Troutman, 1986; de Haan et al., 2013; Murdock, 2013; Teti & Gelfand, 1991; Troutman et al., 2012).

Furthermore, existing reviews on factors associated with PSE have focused on specific populations (e.g., parents and /or children suffering from health problems) (Raynor, 2013), or a specific developmental stage of children (e.g., infant, toddler) (Leahy‐Warren & McCarthy, 2011). Besides, most of the relevant literature has focused mainly on mothers or has not examined gender differences, even though studies have shown that parental gender plays an important role in daily parenting (Giallo et al., 2013; Gordo et al., 2018; Knauth, 2000; Leerkes & Burney, 2007; Salonen et al., 2009; Sevigny & Loutzenhiser, 2010).

### 2.1 Theoretical framework

A process model of parenting was proposed by Belsky (1984). This process model describes how factors from three domains can impact parenting: parental (e.g. developmental history, personality traits and psychological functioning), child (e.g. temperament, child behaviour) and socio‐contextual (e.g. social network, marital quality, employment). Interplay between factors in and between these domains is possible (Belsky, 1984). This model has been widely used in parenting‐related studies (Morse, 2010; Sevigny & Loutzenhiser, 2010; Taraban & Shaw, 2018).

## 3 THE REVIEW

### 3.1 Aims

The aim of this systematic review is to provide an overview of the results of quantitative studies on the parental, child and socio‐contextual factors associated with general PSE among parents with children aged 0–18 years in the general population.

### 3.2 Design

#### 3.2.1 Registration

The systematic review protocol was registered at PROSPERO (registration number: RD42019126737; URL*:* https://www.crd.york.ac.uk/PROSPERO/display_record.php?RecordID=126737 *)*.

### 3.3 Search methods

In January 2019, a systematic literature search was conducted to identify relevant studies published after January 1980. An update of the search was then performed in June 2020. Articles were collected from electronic search engines and through a manual search based on reference articles. The following databases were included in the search: PsycInfo Ovid, MEDLINE Ovid, EMBASE and Web of Science. Combinations of the following keywords were used: “parenting”, “self‐efficacy”, “competence”, “confidence”, “determinant”, “predictor”, “socioeconomic factors” and “demography”. Often used synonyms for PSE were also included: “confidence”, “competence” and parental “self‐esteem” (Vance & Brandon, 2017). The search strategy was adapted to each database, presented in Supplementary file 1.

### 3.4 Inclusion and exclusion criteria

Inclusion and exclusion criteria were set to identify studies reporting associations between various factors with PSE in parents of children aged 0‐18 years old in the general population.

The following inclusion criteria were used: (a) peer‐reviewed article, (b) article published in English, (c) the study reported the association between at least one possible factor and PSE; PSE was reported as the outcome or mediator, (d) the study reported general level PSE; and (e) the study was performed among parents with children aged 0–18 years old from a general population sample. In relation to inclusion criteria number 4, studies sometimes used an alternative term to describe PSE such as parental confidence, self‐agency or self‐definition (Vance & Brandon, 2017). In this review, studies were included when they provided a definition of this alternative term in line with the definition of general level PSE (Jones & Prinz, 2005). Studies that did not provide a clear definition, but used a valid instrument to assess general level PSE as reported by Črnčec (Črnčec et al., 2010) and Wittkowski (Wittkowski et al., 2017), were also included.

Exclusion criteria were (a) the study was performed among parents at risk (e.g., parents/child with certain diseases or impairments), (b) the study included homogeneous subsamples of the population (e.g., only parents from low‐income families), (c) the study described the development of instruments to measure PSE and (d) qualitative studies, review articles, thesis, conference papers and book chapters.

### 3.5 Search outcomes

All references were exported and managed using Endnote X9. Title/abstract screening was performed by two reviewers independently using the abovementioned criteria.

**FIGURE 1:**
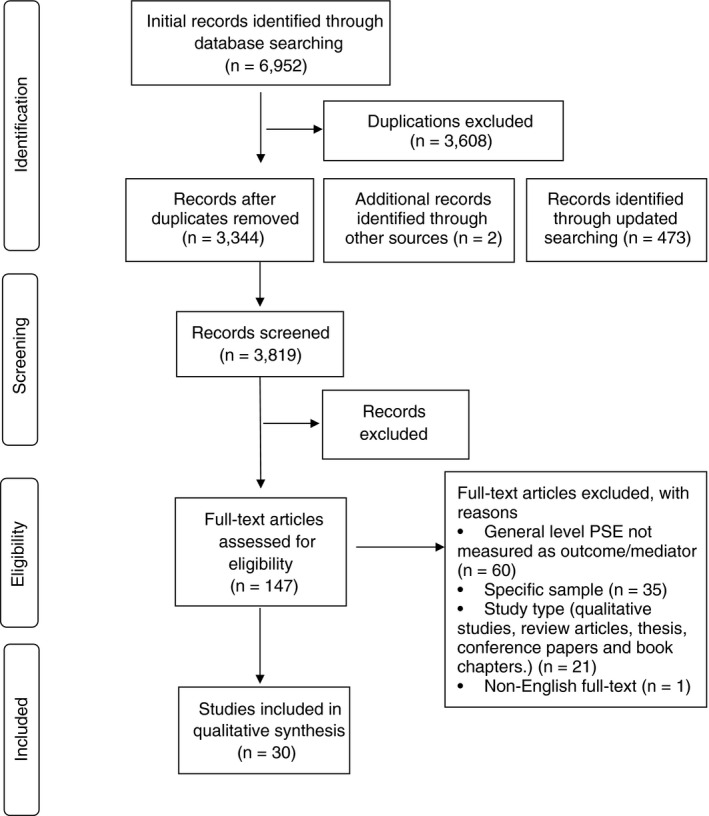
Flow chart for the selection of studies

Relevant articles were retrieved for full‐text reading and further review by two reviewers (YF&MB). Status (included/excluded), study details (first author, year of publication, country), and reasons for exclusion were recorded in a designed access file. Disagreements were discussed with a third reviewer (AG/DW) until consensus was reached. The initial database and manual searches resulted in 3,344 unique titles without duplicate publications; and the updated search yielded 473 unique titles. In total, 147 full‐text articles were identified, of which 30 unique studies met the inclusion criteria. A summary flow chart of the process of literature selection is presented in Figure 1.

#### 3.5.1 Quality appraisal

The methodological quality of the included articles was assessed using the Standard Quality Assessment Criteria for Evaluating Primary Research Papers from a Variety of Fields (QualSyst) (Kmet et al., 2004). The QualSyst is a 14‐item tool that allows for methodological and bias assessment in quantitative and qualitative studies with varying study designs. Because of the observational design of the studies included in this review, item 5 (random allocation), 6 (blinding of investigators) and 7 (blinding of subjects) were removed from the QualSyst. Each item on the QualSyst received a score ranging from 0 to 2 to indicate whether the study fulfilled a criterion (0 = no, 1 = partially, and 2 = yes). All scores were added up to create a total score. The total sum score was then converted into a percentage score (i.e. study total sum score divided by the total possible score of 22) and rated as “excellent” (scores of > 80%), “good” (70%–79%), adequate (55% ‐ 69%) and “low” (<55%) (Castellucci et al., 2020). Two reviewers (YF&MB) assessed quality independently. Disagreements were resolved (AG/DW) via discussion until consensus was reached.

### 3.6 Data extraction

Data from individual study were extracted and organized using an extraction form by one reviewer (YF) and then verified by another reviewer (MB). The extracted information included: first author, year of publication, study country, study design, population and characteristics, including sample size and demographic information, PSE instruments used, type and group (parental, child, socio‐contextual) of the studied factors, and the reported associations between the studied factors and PSE.

From cross‐sectional studies, the reported association between the factors and PSE at the same time point was extracted. From cohort studies, the association between the factors at baseline and PSE at the last follow‐up was extracted. The associations between the studied factors and PSE were represented with “+” for a significant positive association, “−” for a significant negative association, and “0” for a null association. In studies with both univariate and multivariate results, the results from the multivariable associations were included when possible, otherwise the univariate results were used.

### 3.7 Data synthesis

**FIGURE 2:**
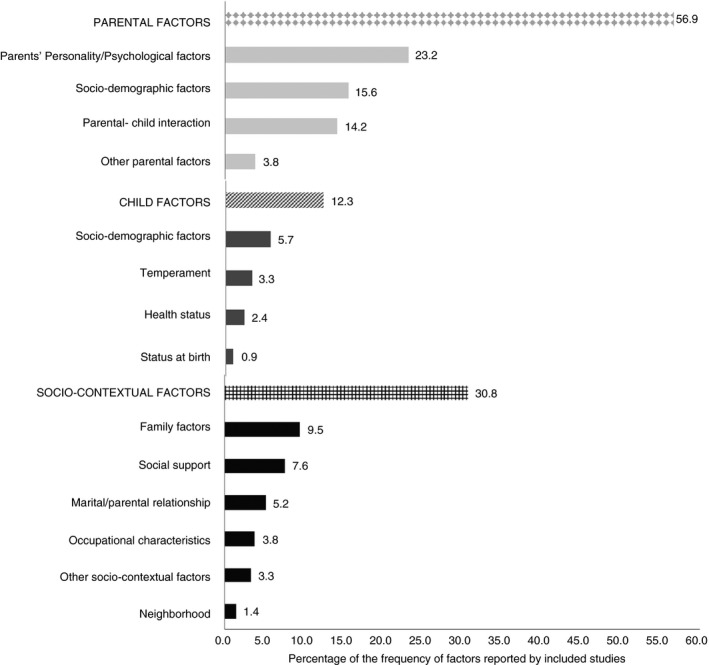
Distribution of the factors associated with PSE among parents of children aged 0–18 years old in the general population

Non‐quantitative data synthesis was performed to summarize the evidence for an association of factors with PSE. Following Belsky's process model, factors were organized into three groups: parental, child and socio‐contextual factors. The factors in each group were further divided into subgroups (Figure 2).

The level of evidence was summarized per factor using a previously established method (Mazarello Paes et al., 2015). The number of studies that reported the association of a specific factor with PSE was divided by the total number of studies that examined that factor. An association between a factor and PSE that was reported by 0%–33%, 34%–59% and 60%–100% of individual studies, was represented using the labels: ‘0’ for no association, ‘?’ for an indeterminate/possible association, ‘+’ for a positive association and ‘−’ for a negative association. Double signs (i.e. ‘00’, ‘??’, ‘++’ and ‘−−’) were given if the association between a factor and PSE was reported by four or more studies.

### 3.8 RESULTS

#### 3.8.1 Characteristics of the included studies

The characteristics of the included studies are summarized in Table 1. Detailed information of the included studies can be found in Table S1. Three studies (10.0%) were published before the year 2000; six studies (20.0%) were published between 2000 and 2009, and twenty‐one studies (70.0%) were published after 2010. Nearly half of the studies were conducted in North America (n = 14, 46.7%). The other studies were carried out in Europe (n = 6, 20.0%), Asia (n = 7, 23.3%) and Australia (n = 3, 10.0%). In total, 18n (60.0%) studies reported results using a cross‐sectional design and 12 (40.0%) studies reported results from longitudinal studies.

**TABLE 1:** Characteristics of the studies included in the systematic review (N = 30)

Characteristics	N	Percentage
		(%)
Study design		
Cross‐sectional	18	60.0
Longitudinal	12	40.0
Year of publication		
<2000	3	10.0
2000–2009	6	20.0
>=2010	21	70.0
Study population		
Mothers only	17	56.7
Fathers only	2	6.7
Both^a^	6	20.0
Parents^b^	5	16.7
Location		
North America	14	46.7
Europe	6	20.0
Asia	7	23.3
Australia	3	10.0
Age period^c^		
Infant (0–1 y)	9	30.0
Pre‐school age (1–4 y)	9	30.0
School age (4–12 y)	8	26.6
School age (12–18 y)	2	6.7
Not specific	2	6.7
Measurement used		
Parenting sense of competence (PSOC) (Johnston & Mash, 1989)	20	66.7
Parenting Stress Index‐ Competence subscale (Abidin, 1997)	4	13.3
Parenting Self‐Agency (PSA) (Dumka, Stoerzinger, Jackson, & Roosa, 1996)	1	3.3
Mother and Baby Scale (Brazelton & Nugent, 1995)	1	3.3
Parental Confidence Index (Mazarello Paes et al., 2015)	1	3.3
Self‐perception of parental role questionnaire (MacPhee, Fritz, & Miller‐Heyl, 1996)	1	3.3
Other PSE measurements (Holloway, Suzuki, Yamamoto, & Behrens, 2005; Suzuki, Holloway, Yamamoto, & Mindnich, 2009)	2	6.7

^a^ Parents were included in the study and subgroup analysis were performed to analyse associations for mothers and fathers separately.

^b^ Parents were included in the study and no subgroup analysis for mothers and fathers were performed

^c^ Based on the mean age of children; two studies: Henney, 2016 and Davidson Arad, McLeigh, & Katz, 2018 only reported range of the children these two studies were categorized into the not specific age group.

The sample sizes ranged from 33 to 1,750. Parents’ ages ranged from 16 to 61 years old. Children's ages ranged from 0 to 18 years old, and the majority of children were between 0–6 years old (n = 22, 73.3%). Half of the studies (n = 17, 56.7%) were performed in a sample of only mothers from the general population, two (6.7%) studies were among fathers only. Eleven studies (36.7%) included both mothers and fathers, and five of them did not examine gender differences.

The most frequently used measurement for PSE was the Parenting Sense of Competence Scale (PSOC, n = 20) (Johnston & Mash, 1989), followed by the Parenting Stress Index (PSI)‐Competence subscale (Abidin, 1997) (n = 4), Parenting Self‐Agency (PSA, n = 1)(Dumka et al., 1996) , Mother and Baby scale (MABS) (n = 1) (Brazelton & Nugent, 1995), Parental Confidence Index (n = 1) (Henney, 2016) and Self‐perception of parental role questionnaire (SPPR)‐Competence subscale (n = 1) (MacPhee et al., 1996). Two studies employed self‐made PSE assessment tools (Holloway et al., 2005; Suzuki et al., 2009).

### 3.9 Quality of the included studies

Scores from the QualSyst checklist ranged from 50.0% to 90.9%, with a mean score of 74.7 ± 9.6% (Table S2). Of the 30 included studies, 12(40.0%) were of excellent quality, 8(26.7%) of good quality, 9(30.0%) of adequate quality and 1(3.3%) of low quality.

### 3.10 Associations between factors and PSE

Results for mothers, fathers and parents (i.e. irrespective of gender) are presented according to the process model of parenting (Table 2). In total 89 factors were reported. Hereof, 74.2% (n = 66) of the factors were reported by one or two studies, 5.6% (n = 5) of the factors were reported by three studies, and 20.2% (n = 18) of the factors were reported by four or more studies. The most frequently studied factors were parental factors, followed by socio‐contextual factors and child factors (Figure 2). In the current study, we mainly reported the level of evidence for the factors that were reported by three or more studies.

**TABLE 2:** Associations between factors and general parenting self‐efficacy in the general population of parents with children between 0 and 18 years, reported by studies included in this review (n = 30)

	Direction of associations^a^					
	Negative (‐)	Null (0)		Positive (+)	n/N^b^	Summary^c^
**MOTHERS**						
**PARENTAL FACTORS**						
**Socio‐demographic factors**						
Age		Suzuki et al., 2009; Troutman et al., 2012; Cutrona & Troutman, 1986; Ercegovac et al., 2013; de Haan et al., 2013; Katkic et al., 2017		Shrooti et al., 2016	1/7	00
Educational level (higher)		Suzuki et al., 2009; Holloway et al., 2005; Hill & Tyson, 2008; Ercegovac et al., 2013; Katkic et al., 2017		Teti & Gelfand, 1991; Cutrona & Troutman, 1986; Shrooti et al., 2016;	3/8	??
Ethnicity		Hill & Tyson, 2008 (African American vs. European American); Murdock, 2013 (white vs. non‐white);		Henney, 2016 (black vs. non‐black)	1/3	0
**Parents' Personality/Psychological factors**						
Anxiety	Jover et al., 2014	Ogel‐Balaban & Altan, 2020			1/2	?
Depression	Teti & Gelfand, 1991; Cutrona & Troutman, 1986; Jover et al., 2014; Gordo et al., 2018	Hill & Tyson, 2008; Baker et al., 2013; Hurwich‐Reiss & Watamura, 2019			4/7	‐‐
Fatigue	Studts et al., 2019	Dunning & Giallo, 2012			1/2	?
Depressed mood		Cutrona & Troutman, 1986			0/1	0
Parenting stress(higher)	Dunning and Giallo, 2012; Gordo et al., 2018; Mazur, 2006	Baker et al., 2013			3/4	‐‐
Parenting rewards	Gordo et al., 2018;				1/1	‐
Parenting stressors	Gordo et al., 2018				1/1	‐
Parenting distress		Mazur, 2006			0/1	0
Self‐efficacy (Global level) (higher)^d^				Murdock, 2013	1/1	+
Parenting self‐efficacy (Task‐specific level) (higher)				Teti & Gelfand, 1991	1/1	+
Partners' parenting self‐efficacy (higher)				Yang et al., 2020	1/1	+
Parental competence (higher)				Knauth, 2000; Gordo et al., 2018;	2/2	+
Self‐esteem (higher)		Baker et al., 2013		Shrooti et al., 2016	1/2	?
Parental affect		Murdock, 2013			0/1	0
Psychological need satisfaction (relatedness)				de Haan et al., 2013	1/1	+
Psychological need satisfaction (autonomy)				de Haan et al., 2013	1/1	+
Personality (Dominance)				Henney, 2016	1/1	+
Personality (Apprehension)	Henney, 2016				1/1	‐
Personality (Rule consciousness)		Henney, 2016			0/1	0
Personality (Perfectionism)		Henney, 2016			0/1	0
Personality (Emotional stability)		Henney, 2016			0/1	0
Personality (Social boldness)		Henney, 2016			0/1	0
Personality (anxiety)	Henney, 2016				1/1	‐
Personality (self‐control)				Henney, 2016	1/1	+
Personality (independence)				Henney, 2016	1/1	+
Perceived Importance for family relationship		Knauth, 2000;			0/1	0
Parental–child interaction						
Parenting quality (Conflict resolution) (higher)				Ercegovac et al., 2013	1/1	+
Parenting quality (Sense of acceptance) (higher)				Ercegovac et al., 2013	1/1	+
Perception of child's vulnerability	Gordo et al., 2018				1/1	+
Parenting behaviour(control)		Murdock, 2013;			0/1	0
Parenting behaviour (hostile or coercive)	Murdock, 2013				1/1	‐
Parenting behaviour (supportive or engaged);		Murdock, 2013			0/1	0
Readiness for pregnancy (ref: unplanned)				Shrooti et al., 2016	1/1	+
Parenting satisfaction (higher)				Gordo et al., 2018; Mazur, 2006; Yang et al., 2020	3/3	+
Parenting discipline (overreactive)	de Haan et al., 2013				1/1	‐
Parenting discipline (warmth)				de Haan et al., 2013	1/1	+
Biased appraisals (negative cognitive error)	Mazur, 2006				1/1	‐
Biased appraisals (positive illusions)		Mazur, 2006			0/1	0
Other parental factors						
Childhood memories (positive)				Holloway et al., 2005; Suzuki et al., 2009	2/2	+
Physical activity				Studts et al., 2019	1/1	+
Parity (non‐primiparous)		Cutrona & Troutman, 1986; Suzuki et al., 2009		Troutman et al., 2012; Shrooti et al., 2016	2/4	??
**CHILD FACTORS**						
**Socio‐demographic factors**						
Age		Suzuki et al., 2009; Holloway et al., 2005; Murdock, 2013; de Haan et al., 2013; Katkic et al., 2017; Studts et al., 2019			0/6	00
Gender (girls)		Holloway et al., 2005; Murdock, 2013; Katkic et al., 2017		de Haan et al., 2013	1/4	00
General Health status (better)		Baker et al., 2013			0/1	0
Child developmental difficulty(yes)				Baker et al., 2013	1/1	+
Behaviour problems	Murdock, 2013; Studts et al., 2019				2/2	‐
Aggression (more)	de Haan, 2013				1/1	‐
Temperament (difficult)	Teti & Gelfand, 1991; Cutrona & Troutman, 1986;	Baker et al., 2013			2/3	‐
Irritable (more)		Troutman et al., 2012			0/1	0
Gestational week	Baker et al., 2013				1/1	+
**SOCIAL CONTEXTUAL FACTORS**						
**Social Support**						
Perceived level of social support (higher)		Baker et al., 2013; Katkic et al., 2017		Cutrona & Troutman, 1986; Shrooti et al., 2016; Teti & Gelfand, 1991	3/5	++
Number of support persons (more)				Shrooti et al., 2016	1/1	+
Source of support		Holloway et al., 2005			0/1	0
Spouses' support				Holloway et al., 2005; Suzuki et al., 2009	2/2	+
Social support satisfaction(higher)		Holloway et al., 2005			0/1	0
Friends support satisfaction (higher)				Suzuki et al., 2009	1/1	+
**Marital /paternal relationship**						
Marital quality (higher)				Katkic et al., 2017	1/1	+
Age at marriage				Shrooti et al., 2016	1/1	+
Number of years married		Cutrona & Troutman, 1986			0/1	0
Marital status (single)		Dunning & Giallo, 2012; Murdock, 2013		Ercegovac et al., 2013	1/3	00
Partner violence (coercive control)		Gou et al., 2019			0/1	0
**Occupation Characteristics**						
Employment (yes)		Dunning & Giallo, 2012; Ercegovac et al., 2013; Katkic et al., 2017		Shrooti et al., 2016	1/4	00
Occupational prestige		Hill & Tyson, 2008			0/1	0
**Family factors**						
Household income (higher)		Murdock, 2013;		Teti & Gelfand, 1991; Shrooti et al., 2016	2/3	+
Number of children (more)		Baker et al., 2013; Holloway et al., 2005; Katkic et al., 2017		Troutman et al., 2012; Ercegovac et al., 2013	2/5	??
Spouses' Employment prestige		Hill & Tyson, 2008;			0/1	0
Spouses’ educational level (higher)		Hill & Tyson, 2008			0/1	0
Economic status (lower)	Hurwich‐Reiss & Watamura, 2019	Troutman et al., 2012; Dunning & Giallo, 2012			1/3	0
Family functioning(better)				Knauth, 2000	1/1	+
Family size (bigger)				Jover et al., 2014; Ercegovac et al., 2013	2/2	+
Family stress (higher)		Hill & Tyson, 2008			0/1	0
Satisfaction with life (higher)		Baker et al., 2013			0/1	0
**Neighbourhood**						
Neighbourhood quality(higher)		Hill & Tyson, 2008			0/1	0
Neighbourhood safety		Hill & Tyson, 2008			0/1	0
Neighbourhood social involvement (higher)		Hill & Tyson, 2008			0/1	0
**Other socio‐contextual factors**						
Religion		Shrooti et al., 2016			0/1	0
Region		Holloway et al., 2005; Shrooti et al., 2016; Ercegovac et al., 2013; Shrooti et al., 2016			0/4	0
Country of birth				Suzuki et al., 2009 (Janpan > USA)	1/1	
**FATHERS**						
**PARENTAL FACTORS**						
**Socio‐demographic factors**						
Age		de Haan et al., 2013			0/1	0
Educational level (higher)		McBride, 1989;		Kwok & Li, 2015	1/2	?
Ethnicity		Murdock, 2013(white vs. non‐white);			0/1	0
**Parents' Personality/Psychological factors**						
Depression	Gordo et al., 2018				1/1	‐
Parenting stress (higher)	Gordo et al., 2018; McBride, 1989;	Kwok & Li, 2015			2/3	‐
Parenting rewards	Gordo et al., 2018				1/1	‐
Parenting stressors	Gordo et al., 2018				1/1	‐
Self‐efficacy (Global level) (higher)				Murdock, 2013;	1/1	+
Partner's parenting self‐efficacy (higher)				Yang et al., 2020	1/1	+
Parental competence (higher)				Gordo et al., 2018; Knauth, 2000	2/2	+
Parental affect		Murdock, 2013			0/1	0
Psychological need satisfaction (relatedness)				de Haan et al., 2013	1/1	+
Psychological need satisfaction (autonomy)				de Haan et al., 2013	1/1	+
Perceived Importance for family relationship		Knauth, 2000			0/1	0
**Parental–child interaction**						
Beliefs on parental role		Kwok & Li, 2015			0/1	0
Involvement		Kwok & Li, 2015			0/1	0
Perception of child's vulnerability				Gordo et al., 2018	1/1	+
Parenting behaviour(control)		Murdock, 2013;			0/1	0
Parenting behaviour (hostile or coercive)		Murdock, 2013			0/1	‐
Parenting behaviour (supportive or engaged);				Murdock, 2013	1/1	+
Parenting satisfaction (higher)				Gordo et al., 2018; Yang et al., 2020	2/2	+
Parenting discipline (overreactive ),	de Haan et al., 2013				1/1	‐
Parenting discipline (warmth)				de Haan et al., 2013	1/1	+
**CHILD FACTORS**						
Age		Murdock, 2013; de Haan et al., 2013; McBride, 1989			0/3	0
Gender (girls)		Murdock, 2013; McBride, 1989		de Haan et al., 2013	1/3	00
General Health status (better)				Salonen	1/1	+
Behaviour problem		Murdock, 2013			0/1	0
Aggression (more)	de Haan et al., 2013				1/1	‐
**SOCIAL CONTEXTUAL FACTORS**						
**Social Support**						
Financial Support				Kwok & Li, 2015	1/1	‘+
Number of support persons (more)		Kwok & Li, 2015			0/1	0
Spouses' support				Kwok & Li, 2015	1/1	+
**Marital /paternal relationship**						
Number of years married		Kwok & Li, 2015			0/1	0
Marital status (single)		Murdock, 2013; Kwok & Li, 2015;			0/2	0
Partner violence (coercive control)	Gou et al., 2019				1/1	‐
Parenting alliance				Kwok & Li, 2015	1/1	+
**Occupation Characteristics**						
Employment (yes)		Kwok & Li, 2015;			0/1	0
**Family factors**						
Household income (higher)		Murdock, 2013; McBride, 1989; Kwok & Li, 2015			0/3	0
Number of children (more)		McBride, 1989			0/2	0
Spouses' Employment (yes)		McBride, 1989			0/1	0
Spouses' income (higher)				Kwok & Li, 2015	1/1	0
Family functioning (better)		Knauth, 2000			0/1	?
Family size (bigger)		Kwok & Li, 2015;			0/1	0
**PARENTS**						
**PARENTAL FACTORS**						
**Socio‐demographic factors**						
Gender(female)			Davidson Arad et al., 2018	de Haan et al., 2009; Cooklin et al., 2012	2/3	?
Age			de Haan et al., 2009; Cooklin et al., 2012		0/2	0
Educational level (higher)			de Haan et al., 2009; Cooklin et al., 2012; Davidson Arad et al., 2018		0/3	0
**Parents' Personality/Psychological factors**						
Anxiety				Giallo et al., 2013	1/1	+
Depression	Giallo et al., 2013				1/1	‐
Fatigue	Cooklin et al., 2012		Giallo et al., 2013; Davidson Arad et al., 2018		1/3	0
Parenting stress (higher)	Giallo et al., 2013				1/1	‐
Sense of Hope				Davidson Arad et al., 2018	1/1	+
Tolerance			Davidson Arad et al., 2018		0/1	0
Personality (autonomy)				de Haan et al., 2009	1/1	+
Personality (agreeableness)				de Haan et al., 2009	1/1	+
Personality (conscientiousness)			de Haan et al., 2009		0/1	0
Personality (extraversion)				de Haan et al., 2009	1/1	+
Personality (emotional stability)				de Haan et al., 2009	1/1	+
Coping strategy (active coping)				Cooklin et al., 2012	1/1	+
Coping strategy (using emotional support)			Cooklin et al., 2012		0/1	0
Coping strategy (using instrument support)			Cooklin et al., 2012		0/1	0
Coping strategy (behavioural disengagement)			Cooklin et al., 2012		0/1	0
Coping strategy (positive reframing)				Cooklin et al., 2012	1/1	+
Coping strategy (planning)				Cooklin et al., 2012	1/1	+
Coping strategy (humour)			Cooklin et al., 2012		0/1	0
Coping strategy (acceptance)			Cooklin et al., 2012		0/1	0
Coping strategy (self‐blame)	Cooklin et al., 2012				1/1	‐
Parenting quality (Conflict resolution)				de Haan et al., 2009	1/1	+
Involvement (more)				Giallo et al., 2013	1/1	+
Perception of child's vulnerability			Cooklin et al., 2012		0/1	0
Parenting satisfaction (higher)				Davidson Arad et al., 2018	1/1	+
Experience with children	Cooklin et al., 2012				1/1	‐
**Others parental factors**						
General Health Status (better)			Giallo et al., 2013; Cooklin et al., 2012; Davidson Arad et al., 2018		0/2	0
**CHILD FACTORS**						
Behaviour problems	Finzi‐Dottan et al., 2011				1/1	−
Emotional Intelligence (higher)				Finzi‐Dottan et al., 2011	1/1	+
Temperament (difficult)	Giallo et al., 2013				1/1	−
**SOCIAL CONTEXTUAL FACTORS**						
**Social Support**						
Perceived support need (higher)	Giallo et al., 2013;Cooklin et al., 2012				2/2	−
Perceived level of social support (higher)				s	1/1	+
**Marital /paternal relationship**						
Marital status (single)		Cooklin et al., 2012			0/1	0
Marital quality (higher)		Giallo et al., 2013			0/1	0
**Occupation Characteristics**						
Employment (yes)		Cooklin et al., 2012; Davidson Arad et al., 2018			0/2	0
**Family factors**						
Economic status (lower)		Cooklin et al., 2012		Davidson Arad et al., 2018	1/2	?
Family size (bigger)		Davidson Arad et al., 2018			0/1	0
Quality of life (higher)				Davidson Arad et al., 2018	1/1	+
**Neighbourhood**						
Neighbourhood collective efficacy (higher)		Davidson Arad et al., 2018			0/1	0
**Other socio‐contextual factors**						
Religion		Davidson Arad et al., 2018			0/1	0

^a^ Summarized data from all studies included in the review.

^b^ n represents the number of studies reporting a significant association, N represents the total number of studies investigating the association.

^c^ The association was labelled as ‘0’ (no association), ‘?’ (indeterminate/possible) and ‘+’ or ‘−’(significant positive/negative association) if supported by 0%–33%, 34%–59% and 60%–100% of individual studies, respectively. In addition, double signs (‘00’, ‘??’, ‘++’ and ‘−−’) were used to indicate if the factors were evaluated by 4 or more studies.

^d^ Global self‐efficacy refers to a person's beliefs about being capable or confident to complete any given task, and parenting is one of these tasks. narrow‐domain PSE concentrates on parental perceived competence in one parenting domain; task‐specific PSE refers to the confidence a parent has over a set of discrete parenting tasks.

### 3.11 Factors associated with PSE: Parental factors

Forty‐eight parental factors were identified, of which 37 factors were only studied in one or two studies.

#### 3.11.1 Mothers

Studies among mothers showed evidence for a negative association of maternal depression (4/7) and parenting stress (3/4) with PSE. Higher maternal satisfaction towards parenting (3/3) was shown to be associated with higher PSE. There was inconsistent evidence for a positive association between educational level (3/8) and parity (2/4) and PSE. There was no evidence for an association between maternal ethnicity (1/3), age (1/7) and PSE. Two studies found a positive association between positive maternal childhood development history and maternal PSE (Holloway et al., 2005; Suzuki et al., 2009). Regarding other maternal personality and psychological factors (24/26) studied, nine positive (de Haan et al., 2013; Henney, 2016; Knauth, 2000; Murdock, 2013; Teti & Gelfand, 1991; Yang et al., 2020), three inconsistent (Baker et al., 2013; Dunning & Giallo, 2012; gel‐Balaban & Altan, 2020; Shrooti et al., 2016), four negative (Gordo et al., 2018; Henney, 2016) and eight null associations (Cutrona & Troutman, 1986; Henney, 2016; Knauth, 2000; Mazur, 2006; Murdock, 2013) with PSE were observed. Evidence for factors related to maternal‐child interaction was inconsistent and reported by a single study only (Ercegovac et al., 2013; Gordo et al., 2018; de Haan et al., 2013; Mazur, 2006; Murdock, 2013; Salonen et al., 2009; Shrooti et al., 2016).

#### 3.11.2 Fathers

Studies among fathers showed evidence for a negative association between paternal parenting stress (2/3) and PSE. For the remaining paternal factors (22/26), nine positive (Gordo et al., 2018; de Haan et al., 2013; Knauth, 2000; Murdock, 2013; Yang et al., 2020), six negative (Gordo et al., 2018; de Haan et al., 2013; Kwok & Li, 2015; McBride, 1989), six null (Knauth, 2000; Kwok & Li, 2015; McBride, 1989; Murdock, 2013) and one inconsistent (Salonen et al., 2009) associations were reported.

#### 3.11.3 Parents

Studies among parents showed inconsistent evidence that mothers have higher PSE (2/3). There was no evidence for associations of parental educational level (0/3), fatigue (1/3) with PSE. For the remaining parental factors (24/27); 12 positive (Cooklin et al., 2012; Davidson Arad et al., 2018; Giallo et al., 2013; de Haan et al., 2009), four negative (Cooklin et al., 2012; Giallo et al., 2013) and 10 null (Cooklin et al., 2012; Davidson Arad et al., 2018; Giallo et al., 2013; de Haan et al., 2009) associations with PSE were reported.

### 3.12 Factors associated with PSE: Child characteristics

Nine child factors were identified, of which three factors were only studied in one or two studies.

#### 3.12.1 Mothers

For mothers, there was evidence for a negative association between difficult child temperament (2/3) and PSE. There was no evidence for an association between child age (0/6), child gender (1/4) and maternal PSE. Among other child characteristics, two positive(Baker et al., 2013; Katkic et al., 2017),two negative (de Haan et al., 2013; Murdock, 2013; Studts et al., 2019) and two null (Salonen et al., 2009; Troutman et al., 2012) associations were reported.

#### 3.12.2 Fathers

For fathers, there was no evidence of an association between child age (0/3), child gender (1/3) and paternal PSE. Evidence for other factors related to child characteristics and paternal PSE was inconsistent and studied by single studies.

#### 3.12.3 Parents

For parents, there was evidence that parents of children with less behavioural problems, easier temperament and more emotional intelligence have higher PSE. However, these findings were only studied by two studies (Finzi‐Dottan et al., 2011; Giallo et al., 2013).

#### 3.12.4 Factors associated with PSE: Socio‐contextual factors

Thirty‐two socio‐contextual factors were identified, of which 24 factors were only studied in one or two studies.

#### 3.12.5 Mothers

For mothers, there was consistent evidence that mothers with a higher household income (2/3) and a higher perceived level of social support (3/5) have higher PSE. There was inconsistent evidence available for a positive association between the number of children (2/5) and maternal PSE. There was no evidence for an association between employment status (1/4), marital status (1/3), economic status (1/3) and maternal PSE. Three studies investigated the association between different sources of social support and satisfaction towards support, with three positive (Holloway et al., 2005; Shrooti et al., 2016; Suzuki et al., 2009) and two null (Holloway et al., 2005) associations with PSE reported. Of the 4/6 factors studied related to marital relationship and PSE, two positive (Shrooti et al., 2016) (Katkic et al., 2017)and two null‐associations (Cutrona & Troutman, 1986; Gou et al., 2019) were reported. One study reported a null association between occupational prestige and PSE (Hill & Tyson, 2008). Regarding family factors, there was some evidence that better family health (1/1) and larger family size (2/2) were associated with higher PSE. And there was no evidence of an association between PSE and other family factors (Baker et al., 2013; Dunning & Giallo, 2012; Hill & Tyson, 2008; Hurwich‐Reiss & Watamura, 2019; Troutman et al., 2012). One study found no associations between neighbourhood factors and PSE (Hill & Tyson, 2008).

#### 3.12.6 Fathers

For fathers, there was no evidence of an association between household income (0/3) and paternal PSE. Evidence for other factors related to social support, marital status (Kwok & Li, 2015), occupation (Mazur, 2006), family (Knauth, 2000; Kwok & Li, 2015; McBride, 1989; Salonen et al., 2009) and PSE was inconsistent. Most of these factors were reported by one study (Table 2).

#### 3.12.7 Parents

For parents, evidence for factors related to social support, marital status, occupation, family, neighbourhood and PSE was reported by one or two studies; two positive (Davidson Arad et al., 2018; Finzi‐Dottan et al., 2011), one negative (Cooklin et al., 2012; Giallo et al., 2013), one inconsistent (Cooklin et al., 2012; Davidson Arad et al., 2018) and six null‐ (Cooklin et al., 2012; Davidson Arad et al., 2018; Giallo et al., 2013) associations with PSE were found.

## 4 DISCUSSION

With this systematic review, we aimed to provide an overview of the available literature on factors associated with general parenting self‐efficacy (PSE) among parents of children aged 0–18 years old in the general population. In total, 30 studies were included. Overall, the vast majority of the studies was performed among mothers only and followed a cross‐sectional design. Across studies a broad range of parental, child and social‐cultural factors was evaluated in relation to PSE. Consequently, the evidence synthesis in this review was often limited to the fact that each factor was studied only in a small set of studies. Given these methodological considerations, this review concludes that based on the included studies, there is an association between parenting stress, depression, child temperament, household income, perceived social support and PSE. For the factors parental age, ethnic‐background, employment, marital status, economic status, child age and child gender no association with PSE was evident. Inconsistent findings were reported for an association between parental educational level, parity, number of children living in the household and PSE.

### 4.1 Parental factors and PSE

According to the process model of parenting, parental factors, compared with child and socio‐contextual factors, may have the strongest impact on parenting behaviours; impacting parenting both directly and through social networks and the children (Belsky, 1984). In this review, parental factors were studied in over 50% of the studies (Baker et al., 2013; Cutrona & Troutman, 1986; Dunning & Giallo, 2012; Ercegovac et al., 2013; Gordo et al., 2018; Gou et al., 2019; de Haan et al., 2013; Henney, 2016; Holloway et al., 2005; Jover et al., 2014; Knauth, 2000; Kwok & Li, 2015; Mazur, 2006; McBride, 1989; Murdock, 2013; Salonen et al., 2009; Shrooti et al., 2016; Suzuki et al., 2009; Troutman et al., 2012). Specifically, the parental demographic characteristics education level and ethnic background have been suggested to have a strong impact on PSE (Cutrona & Troutman, 1986; Dunning & Giallo, 2012; Ercegovac et al., 2013; Gordo et al., 2018; Gou et al., 2019; de Haan et al., 2013; Henney, 2016; Hill & Tyson, 2008; Holloway et al., 2005; Kwok & Li, 2015; McBride, 1989; Murdock, 2013; Salonen et al., 2009; Suzuki et al., 2009; Teti & Gelfand, 1991; Troutman et al., 2012). In the current review, three studies reported positive association between a higher education level and higher PSE, whilst five studies reported a null association (Cutrona & Troutman, 1986; Ercegovac et al., 2013; Hill & Tyson, 2008; Holloway et al., 2005; Salonen et al., 2009; Shrooti et al., 2016; Suzuki et al., 2009; Teti & Gelfand, 1991). Seo et al (Seo, 2006) have suggested that highly educated parents may actively obtain knowledge on parenting and may perceive more social support compared with lower educated parents, and in turn could be more confident in their role of parenting. More in line with our findings, they also argued that compared with highly educated parents, less educated parents could perceive less complexities in parenting, and thus are more confident in their role in parenting (Seo, 2006).

With regard to ethnic background, previous literature has reported cross‐cultural differences in PSE (Bornstein et al., 2011; Kiang et al., 2017). In this review three studies included ethnic background as one of the factors under study in relation to general PSE (Henney, 2016; Hill & Tyson, 2008; Murdock, 2013). With regard to studies among mothers, one study reported an association between maternal ethnic background (i.e. Black vs. non‐Black) (Henney, 2016) and general PSE, two studies reported no difference in PSE between African American and European American (Hill & Tyson, 2008), white and non‐white mothers (Murdock, 2013). The assessment of ethnicity in these studied was mainly based on country of birth and immigration status (Henney, 2016; Hill & Tyson, 2008; Murdock, 2013), therefore potentially reflects only part of cultural background (Diversity Council Australia, 2020). Cultural differences in PSE may relate to different attributions, attitudes and beliefs in parenting, which in turn could impact parental confidence in parenting (Bornstein et al., 2011; Kiang et al., 2017; Kwok & Li, 2015). Therefore, the association between ethnic background and PSE might be mediated by aspects of parenting, for example parenting warmth (Hill & Tyson, 2008). More studies are recommended to assess cultural background) and the relation with parenting and PSE.

Besides parental demographic factors, parental psychological factors are reported to impact parenting (Belsky, 1984). Especially, depression and stress are often studied (Wilson & Durbin, 2010). In line herewith, this review observed evidence among included studies for an association between higher maternal depression and lower PSE (Cutrona & Troutman, 1986; Gordo et al., 2018; Jover et al., 2014; Teti & Gelfand, 1991) as well as an association between higher parental stress and lower PSE (Dunning & Giallo, 2012; Gordo et al., 2018; Mazur, 2006; McBride, 1989). It is reasonable to hypothesize that parents who suffer from depression and (or) parenting stress may find parenting more demanding; engaging in daily child‐rearing activities to meet a child's needs can be more challenging than usual (Cooklin et al., 2012; Giallo et al., 2013). The consistent findings for parental depression and stress, underline the importance for health professionals to monitor the health and well‐being of a family to be able to provide appropriate support.

Finally, parental developmental history, e.g. the experiences of parents in their own childhood with regard to their parents’ parenting, has been highlighted as an important factor in shaping parenting (Belsky, 1984). Parenting, or aspects of parenting behaviour, might be transmitted across generations (Belsky et al., 2009). Two studies were identified in this review evaluating the association between childhood memories of parental warmth and support and PSE (Holloway et al., 2005; Suzuki et al., 2009). Both studies reported a positive association between warm childhood memories and PSE. Other aspects of parental developmental history were not reported on but might be relevant for inclusion in future research.

### 4.2 Child factors and PSE

In this review the associations between child factors and PSE were less often studied compared with parental and socio‐contextual factors; 9 (10.1%) out of the 89 studied factors. The child factors most often studied, besides child age, were child behaviour problems and temperament. (Baker et al., 2013; Cutrona & Troutman, 1986; de Haan et al., 2013; Murdock, 2013; Teti & Gelfand, 1991; Troutman et al., 2012). There was evidence for a negative association between a child's difficult temperament and PSE (Baker et al., 2013; Cutrona & Troutman, 1986; de Haan et al., 2013; Teti & Gelfand, 1991). For example, de Haan et al used the Child Behaviour Checklist and observed that parents of children who are more aggressive had lower PSE (de Haan et al., 2013). Murdock et al reported that one‐point increase in problem behaviour total score would lower maternal PSE by 0.34 (*p* < 0.05). These behavioural and temperament characteristics of a child could make certain elements of parenting more challenging, and decrease parenting self‐efficacy. Youth health care providers monitoring child and family well‐being should be aware of potential additional challenges in parenting, for parents of children with a difficult temperament of behaviour problems.

The included studies suggested that there is no association between child age and PSE of the parents. According to previous studies PSE is dynamic as parenting tasks vary by the developmental stage of the child, and parents learn new skills adapting to the changing needs of their children (Bandura, 1977; Jones & Prinz, 2005). Over 70% of the studies included in this systematic review were performed among samples of children between 0 and 6 years and were analysed cross‐sectional (Baker et al., 2013; Cutrona & Troutman, 1986; Dunning & Giallo, 2012; Ercegovac et al., 2013; Giallo et al., 2013; Gordo et al., 2018; Gou et al., 2019; Holloway et al., 2005; Jones & Prinz, 2005; Knauth, 2000; Kwok & Li, 2015; Mazur, 2006; McBride, 1989; Murdock, 2013; Salonen et al., 2009; Shrooti et al., 2016; Suzuki et al., 2009; Teti & Gelfand, 1991; Troutman et al., 2012). Hence, future longitudinal studies across multiple developmental stages of children are recommended to assess the association between child age and PSE.

### 4.3 Socio‐contextual factors and PSE

Belsky suggested that a positive marital relationship is supportive of competent parenting (Belsky, 1984). It is likely that parents can get support and encouragement on parenting from their direct partners, which may help develop, maintain and increase parenting self‐efficacy. We identified nine studies that included marital status or single/non‐single parents, and reported no association with PSE (Cutrona & Troutman, 1986; Dunning & Giallo, 2012; Gou et al., 2019; Kwok & Li, 2015; Murdock, 2013; Salonen et al., 2009; Shrooti et al., 2016). Only one study reported that single mothers had lower PSE (Ercegovac et al., 2013). A social support network may be equally as important for families as a marital relationship (Belsky, 1984). A higher level of social support is a well‐established predictor of optimal parenting practices and parent well‐being (Angley et al., 2015). Parents may get advice and support on child‐rearing from their partner, family, friends and social network, supporting parental perceived PSE. Besides, when feeling supported, parents may also experience less parenting stress and therefore have more confidence in their role of the parent (Angley et al., 2015; Östberg & Hagekull, 2000). The studies in this review suggested a positive association between parent perceived level of support and PSE (Baker et al., 2013; Cutrona & Troutman, 1986; Katkic et al., 2017; Shrooti et al., 2016; Teti & Gelfand, 1991). More specifically studies indicate that apart from perceived social support, the source, content and quality of the support could also be related to PSE (Holloway et al., 2005; Suzuki et al., 2009). Family structures have been becoming more diverse over the past decades (e.g., divorce, co‐habitation, same‐sex marriage has been increasing) (Livingston, 2014), studying family structure and the social relationships in relation to PSE is relevant.

### 4.4 Mothers, fathers and PSE

Previous studies demonstrated that mothers and fathers might perceive their role as a parent differently (Daly, 2002; Schluterman, 2007). Although the studies included in our review were mostly performed in samples of mothers only, there were a few that were performed in mixed samples (Gordo et al., 2018; Gou et al., 2019; de Haan et al., 2013; Knauth, 2000; Murdock, 2013; Salonen et al., 2009). From these studies two main conclusions can be drawn. First of all, studies suggest mixed evidence for a gender difference in overall level of PSE: two studies (Gordo et al., 2018; Salonen et al., 2009) reported mothers having a higher PSE and three studies (Dunning & Giallo, 2012; Gou et al., 2019; de Haan et al., 2013) reported comparable PSE between mothers and fathers. Second, when studying factors associated with PSE, studies suggest some that certain factors are factors associated with PSE in similar directions, for both fathers and mothers (e.g. depression and parenting stress) (Gordo et al., 2018; Gou et al., 2019; de Haan et al., 2013; Knauth, 2000; Murdock, 2013; Salonen et al., 2009). Others factors seem to be differently associated with PSE for fathers and mothers. For example, family functioning was suggested to be associated with maternal PSE only (Knauth, 2000), and parenting stress with paternal PSE (Sevigny & Loutzenhiser, 2010). However, these factors were only reported by one or two studies (Gordo et al., 2018; Gou et al., 2019; de Haan et al., 2013; Knauth, 2000; Murdock, 2013; Salonen et al., 2009) (Table S3). Additional research is recommended to study both maternal and paternal PSE and associated factors.

### 4.5 Methodological considerations

The strengths of this systematic review include the large number of studies identified and included. This was possible by including studies that used alternative terms for PSE (i.e., parenting sense of competence, parenting satisfaction) to identify all relevant published papers. We present a data synthesis of available literature for the associations among mothers and fathers, adding to the existing literature. However, several limitations should also be addressed. First, publication bias cannot be ruled out as only peer‐reviewed papers in the English language were included. This may lead to an under‐ or overestimation of the strength of the observed associations. Second, causalities cannot be ascertained as most of the studies followed a cross‐sectional design. Moreover, a wide range of self‐report PSE measures was used in the included studies. Although these measures are all used to measure PSE, there is a difference in, for example, the exact items used in these measures. Third, studies included were most often performed among samples obtained in developed countries, which may limit the generalizability of the results to other populations.

### 4.6 Directions for future research

Three overall recommendations for future research can be formulated. First, longitudinal studies are recommended to evaluate the associations of factors with PSE over time. These studies could also provide insight in factors associated with PSE for parents of both younger and older children. Thus far, most studies are cross‐sectional by design and focus on parents of children 0–6 years old. For each factor, researchers should consider carefully the concept that is being assessed by which definition (e.g., cultural background or immigration status). Second, parental, child and socio‐contextual factors could interact with each other, or act as mediators or moderators in the association with parenting (Belsky, 1984). In addition, studies have shown that the association between parenting and child development could also be bidirectional (Perry et al., 2014). Researchers are recommended to take these considerations, potentially guided by a theoretical framework, into account when developing the study design. Finally, most of the studies included in this review focused on mothers. In the past decades, fathers have taken on more active roles in caregiving (Lewis & Lamb, 2003). Researchers are recommended to continue efforts to include fathers when studying family health, wellbeing and factors associated with parenting and PSE.

### 4.7 Conclusion

In this study, an overview of the evidence regarding the association of parental, child and socio‐contextual factors with PSE among parents of children aged 0–18 years old in the general population is provided. A range of factors studied in relation to PSE was identified in this systematic review. However, the majority of the factors was reported by one or two studies often implementing a cross‐sectional design. There was some evidence for an association between potentially modifiable factors (e.g. parenting stress, depression and perceived social support) and PSE in the general population. This information may be used by health and social professionals supporting child health and well‐being. Future longitudinal studies are recommended to study parental, child and socio‐contextual factors associated with PSE to inform the development of intervention strategies.

## CONFLICT OF INTEREST

No conflict of interest has been declared by the author(s).

## AUTHOR CONTRIBUTIONS

All authors have agreed on the final version. The study was developed by HR and YF. HR, AG, DW and YF designed the methodology for the review. YF and MB contributed to the records screening, data extraction and quality assessment. YF drafted the paper; HR, AG, DW and MB contributed to critical revision of the paper. All authors approved the final version.

### Peer Review

The peer review history for this article is available at https://publons.com/publon/10.1111/jan.14767.

## Supporting information

Supplementary Material

## Data Availability

The data that support the findings of this study are available from the corresponding author upon reasonable request.
