# Supplementary material for: Factors associated with parenting self‐efficacy: A systematic review
Source: J Adv Nurs. 2021 Feb 15;77(6):2641–61. doi: 10.1111/jan.14767 (PMC8248335; doi:10.1111/jan.14767)
Supplement: Supplementary file 1 — Supplementary Material [file JAN-77-2641-s001.docx]

***Supplementary files***

Supplement 1: searching strategies

**embase.com**

((('competence'/de) AND ('child parent relation'/exp OR parent/exp OR 'child rearing'/de OR parenthood/de OR 'parental attitude'/de OR 'parental behavior'/de)) OR (((parent* OR mother* OR father* OR paternal* OR maternal* OR 'child rearing' OR childrearing) NEAR/6 (competenc* OR confiden* OR self-efficac*))):ab,ti) AND ('social determinants of health'/de OR 'determinant'/de OR 'predictor variable'/de OR 'prediction'/de OR forecasting/de OR 'income group'/exp OR 'educational status'/exp OR 'household income'/exp OR 'demography'/exp OR 'neighborhood'/exp OR 'family size'/exp OR 'ethnic group'/exp OR 'employment status'/de OR employment/de OR 'social status'/exp OR (determinant* OR predictor* OR predictive OR forecast* OR prediction* OR ((education* OR income OR socioeconomic* OR socio-economic* OR social) NEAR/3 (status OR level OR group* OR household OR background OR incongru* OR resource*)) OR (Psychosocial* NEAR/3 variable*) OR (economic* NEAR/3 hardship*) OR demograph* OR sociodemograph* OR neighborhood* OR neighbourhood* OR (Financial* NEAR/3 resource*) OR (family NEAR/3 (size OR large OR small)) OR (number NEAR/6 (children OR offspring)) OR ethnic* OR multiethnic* OR employment*):ab,ti) NOT ([Conference Abstract]/lim) AND [English]/lim

**Medline Ovid**

(((Mental Competency/) AND (exp Parent-Child Relations/ OR Child Rearing/ OR exp Parents/)) OR (((parent* OR mother* OR father* OR paternal* OR maternal* OR child rearing OR childrearing) ADJ6 (competenc* OR confiden* OR self-efficac*))).ab,ti.) AND (Social Determinants of Health/ OR Forecasting/ OR Educational Status/ OR Demography/ OR Residence Characteristics/ OR Family Characteristics/ OR Ethnic Groups/ OR Employment/ OR Social Class/ OR (determinant* OR predictor* OR predictive OR forecast* OR prediction* OR ((education* OR income OR socioeconomic* OR socio-economic* OR social) ADJ3 (status OR level OR group* OR household OR background OR incongru* OR resource*)) OR (Psychosocial* ADJ3 variable*) OR (economic* ADJ3 hardship*) OR demograph* OR sociodemograph* OR neighborhood* OR neighbourhood* OR (Financial* ADJ3 resource*) OR (family ADJ3 (size OR large OR small)) OR (number ADJ6 (children OR offspring)) OR ethnic* OR multiethnic* OR employment*).ab,ti.) AND english.la.

**PsycINFO Ovid**

(((Competence/) AND (exp Parent Child Relations/ OR Childrearing Attitudes/ OR Childrearing Practices/ OR exp Parental Attitudes/ OR exp Parents/)) OR (((parent* OR mother* OR father* OR paternal* OR maternal* OR child rearing OR childrearing) ADJ6 (competenc* OR confiden* OR self-efficac*))).ab,ti.) AND (Prediction/ OR Educational Background/ OR Demographic Characteristics/ OR Neighborhoods/ OR Family Background/ OR "Racial and Ethnic Groups"/ OR Employment Status/ OR Social Class/ OR (determinant* OR predictor* OR predictive OR forecast* OR prediction* OR ((education* OR income OR socioeconomic* OR socio-economic* OR social) ADJ3 (status OR level OR group* OR household OR background OR incongru* OR resource*)) OR (Psychosocial* ADJ3 variable*) OR (economic* ADJ3 hardship*) OR demograph* OR sociodemograph* OR neighborhood* OR neighbourhood* OR (Financial* ADJ3 resource*) OR (family ADJ3 (size OR large OR small)) OR (number ADJ6 (children OR offspring)) OR ethnic* OR multiethnic* OR employment*).ab,ti.) AND english.la. NOT (news OR congres* OR abstract* OR book* OR chapter* OR dissertation abstract*).pt.

**Web of science**

TS=(((((parent* OR mother* OR father* OR paternal* OR maternal* OR "child rearing" OR childrearing) NEAR/5 (competenc* OR confiden* OR self-efficac*)))) AND ((determinant* OR predictor* OR predictive OR forecast* OR prediction* OR ((education* OR income OR socioeconomic* OR socio-economic* OR social) NEAR/2 (status OR level OR group* OR household OR background OR incongru* OR resource*)) OR (Psychosocial* NEAR/2 variable*) OR (economic* NEAR/2 hardship*) OR demograph* OR sociodemograph* OR neighborhood* OR neighbourhood* OR (Financial* NEAR/2 resource*) OR (family NEAR/2 (size OR large OR small)) OR (number NEAR/5 (children OR offspring)) OR ethnic* OR multiethnic* OR employment*))) AND DT=(article) AND LA=(english)

| **Supplementary Table 1. Detailed information about the characteristics of the studies included in the systematic review (N=30)** | | | | | | | | | | | | | | | | | | | |
| --- | --- | --- | --- | --- | --- | --- | --- | --- | --- | --- | --- | --- | --- | --- | --- | --- | --- | --- | --- |
| **Author, year** | **Study design** | **Comparison of** | **Country** | **Sample Size** | **Age (years)- parents**  **mean (SD)[range]** | | **Percentage girls (%)** | **Age (years)- children**  **mean (SD)[range] ^†^** | | **Instruments used** | | **Analysis** | | **Parental factors ^‡^** | | **Child factors** | | **Socio-contextual factors** | |
| **Mothers Only Studies** | | | | | |  | | |  | |  | |  | |  | |  | |  |
| Dunning & Giallo, 2012 | CS | / | Australia | 1,022 | 34.51 (4.99)  [18-50] | | 46.9 | 2.59(1.90)  [0-6] | | PSOC-Efficacy subscale | | Path analyses | | Parenting stress (-), Fatigue (0); | | gender (0); | | employment (0); family structure (0): sole or couple; socio-economic status (0) | |
| Ercegovac, Ljubetic, & Pericic, 2013 | CS | / | Croatia | 468 | <30  31-40  >40 | | x | 5.4(3-6.8) | | PSOC | | Oneway ANOVA | | mother' age (0), employment (0), education (0), number of children (+), satisfaction (+), conflict resolution (+), acceptance (+) | |  | | place of residence (-); marital status (ref: single-) parent family +), | |
|  |  |  |  |  |  |  |  |  |  |  |  | correlation  analysis | |  |  |  |  |  |  |
|  |  |  |  |  |  |  |  |  |  |  |  |  | |  |  |  |  |  |  |
| Henney, 2016 | CS | / | America | 121 | 35 (7.8)  [19-54] | | x | [6-18] | | Parental Confidence Index | | regression analysis | | Dominance (+); Apprehension (-) Rule; consciousness (0); Perfectionism (0); Emotional stability (0); Social boldness (0); anxiety (-); self-control (+); independence (+) | |  | | ethnicity (black +) | |
| Holloway, Suzuki, Yamamoto, & Behrens, 2005 | CS | / | Japan | 116 | x | | x | 5.2(0.40)  [5-6] | | self-made | | correlation;  regression analysis | | childhood memories (+), husband support satisfaction (+), relative support satisfaction (0), mothers' education (0), | | child age (0), number of children (0), gender (0) | | Source of support: friends support (0), mothers' mothers' support (0), city of residence (0) | |
| Hurwich-Reiss & Watamura, 2019 | CS | / | America | 127 | 31.64(6.04) | | 43 | 2.18 (0.73) | | PSOC | | regression analysis | | parental depression (0); | |  | | economic pressure (-); | |
|  |  |  |  |  | [18-44] | |  | [0.75-3.67] | |  |  |  |  |  |  |  |  |  |  |
| Katkic, Morovic, & Kovacic, 2017 | CS | child with and without development disabilities (DD) | Croatia | 71  33 mothers of children with DD and 38 mothers of children without DD | 34.29(5.17)  [20-44] | | 38 | children with DD:5.21(3.09); children without DD: 5.34(2.62)  [1-13] | | PSOC | | regression analysis | | maternal age (0); maternal educational level (0); number of children (0); | | child gender (0); child age (0); child developmental difficulty(yes)(+); | | maternal employment status (0); marital quality (+); social support (0) | |
| Mazur, 2006 | CS | / | America | 72 | 33(4.8) | | x | 1.31 (0.32)  [2-5] | | PSOC-Efficacy subscale | | regression analyses | | negative cognitive error (-), positive illusions (0), parenting daily hassles (0), parenting stress (+), parenting distress (0), perceived parenting stress (-), parenting satisfaction (+), | |  | |  | |
| gel-Balaban & Altan, 2020 | CS | use Facebook | Turkey | 332 | 34.55 (5.44)  [20-48] | | x | 5.05(4.27) | | SPPR | | regression analysis | | anxiety (0) | |  | |  | |
|  |  | vs no use |  |  |  |  |  |  |  |  |  |  |  |  |  |  |  |  |  |
| Shrooti, Mangala, Nirmala, Devkumari, & Dharanidhar, 2016 | CS | / | Nepal | 290 | <=19  20-35  >35 | | x | [0-1] | | PSOC | | regression  analysis | | Mothers' age (+), educational level (+), employment (yes +), readiness for pregnancy (planed +), age at marriage (+), per capita income (+), self-esteem (+), parity (non-primiparous +), residence urban (0), religion (0) | |  | | social support (+), number of support persons (+) | |
| Studts, Pilar, Jacobs, & Fitzgerald, 2019 | L, 3w FU | / | America | 137 | 32.0(8.4) | | 46 | [3-5] | | PSOC | | regression analysis | | fatigue (-); physical activity (+); | | child age (0); child disruptive behavior problems (-); | | SES index (0); | |
| Suzuki, Holloway, Yamamoto, & Mindnich, 2009 | CS | Japanese VS American | America | 235  121 American+114 Japanese mothers | Japan 35.61 (3.80) US 38.13 (4.24)  [27-45] | | 52.9(American) VS 50(Japanese) | American:5.03 (4.02); Japanese 6.89 (3.48)  [5-6.12] | | Self-made | | regression analysis | | age (0); education (0); | | age (0); birth order (0); | | country:(Japan: +); childhood parental support (+); satisfaction with husband' support (+); | |
|  |  |  |  |  |  |  |  |  |  |  |  |  |  |  |  |  |  | satisfaction with friends' support (+) | |
| Teti & Gelfand, 1991 | CS | depressed VS no depressed | America | 86  48 depressed VS 38 nondepressed | [16-40] | | x | 0.61  [0.25-1.08] | | PSI-competence scale | | Correlation  analysis | | maternal depression (-); task-specific PSE (+); maternal education (+); family income (+) | | perceived infant difficulty (-); | | social-marital support (+); | |
| Baker, McGrath, Pickler, Jallo, & Cohen, 2013 | L, 6w FU | mothers of preterm infants Vs term infants | America | 70  49 term & 21 late preterm infants’ mothers | 28.2 | | x | [0-0.12] | | PSOC | | Correlation  analysis | | satisfaction with life (0), self-esteem (0), depression/mood (0), stress (0), experience (0), | | late preterm (-), infant wellbeing (0), perceived infant temperament (0), | | support (0), | |
| Cutrona & Troutman, 1986 | L, 3m FU | / | America | 55 | 27.3  [19-38] | | x | 0.25 (0.03) | | PSOC-Efficacy subscale | | correlation;  path analyses | | age (0), number of years married (0), parity (primipara 0), number of years of education (+), mood (0), depression (-), | | infant difficulty (-), | | social support (+) | |
| Hill & Tyson, 2008 | L, | African American (N=54)  European American (49) | America | T1: 103;  T2: 86 | x | | x | Fourth Grade | | PSOC-Efficacy subscale | | regression analysis | | depression (0) | |  | | Ethnicity:(American 0); Family stress (0); Neighborhood quality (0); Neighborhood social involvement (0); Neighborhood collective socialization (0); Neighborhood collective parenting (0); Mother’s education (0); Mother’s occupational prestige (0); Father’s education (0); Father’s occupational prestige (0); | |
| Jover et al., 2014 | L, 8w FU | / |  | T1: 317; | 32.09 (4.44) | | 51.5 | 0.15 | | MBS | | regression analysis | | postpartum depression (-); anxiety (-) | |  | | Number of people at home (>2, +) | |
|  |  |  | Spain | T2: 257 | [18-36] | |  |  |  |  |  |  |  |  |  |  |  |  |  |
| Troutman, Moran, Arndt, Johnson, & Chmielewski, 2012 | L, 16w FU | mothers of irritable and non-irritable infants | USA | 53  irritable 24 & non-irritable 29 | 31[23-42] | | 47.16 | [0.08-1.08] | | PSOC -Efficacy subscale | | ANCOVA (analysis of covariance) | | age (0); Experience with children (+) | | parity (non-primiparous, +); irritable (0) | | seriocomic-status (0); | |
| **Fathers Only Studies** | | | | | | | | | | |  | |  | |  | |  | |  |
| Kwok & Li, 2015 | CS | / | China | 1,750 | 39.09(7.36) | | x | 4.18(1.2)  [2-6] | | PSOC-Efficacy subscale | | Correlation;  path analysis | | age (+), household income (0), education level (+), employment status (0), marital status (0), years of marriage (0), number of children (0), (grand)parents live in house (0), domestic helpers (0), parenting alliance (+), | |  | | spousal support (+), Spousal capital (+), receiving financial support (+), | |
|  |  |  |  |  |  |  |  |  |  |  |  |  |  | marital satisfaction (+), fathers' beliefs on role (0), fathering stress (0), father involvement (0) | |  |  |  |  |
| McBride, 1989 | CS | / | America | 94 | 35.6  [26-46] | | x | 3.5[1.6-4.8] | | PSOC | | Correlation  analysis | | Age(0), income(0), maternal employment(0), Fathers' education(0), parenting stress(+), child domain stress: adaptability(+)acceptability(+), perceptions of children's demandingness(+),moodiness(+),distractibility (+), reinforce(+), parent domain stress: depression(+), attachment to children(+), restriction(+), competence(+), isolation(+), relationship with their spouses(+), parent health(+), | | Age (0),  gender (0),  number of children (0), | |  | |
| **Parents Mixed Studies** | | | | | |  | | |  | |  | |  | |  | |  | |  |
| Cooklin, Giallo, & Rose, 2012 | CS | / | Australia | 1,276 | Mothers:34.3(4.8)  Fathers:36.7(5.5) | | x | [0-5] | | PSOC | | regression analysis | | Fatigue(-), age(0), gender(0), number of child< 5 years (-), Coping strategies: active coping(+), using emotional support(0), using instrument support(0), behavioral disengagement(0), positive reframing(+), planning(+), humor(0), acceptance(0), self-blame(-), physical health(0), | |  | | Education level (0), Relative Socio-Economic Disadvantage: SEIFA index (0), household type(single 0), social support satisfaction(0), social support need(-),employment(0), | |
| Davidson Arad, McLeigh, & Katz, 2018 | CS | / | Israel | 198 | 34.1[20-54] | | x | <10 | | PSOC-Efficacy subscale | | correlation  t test | | perceived health (0), gender (0), work status (0): at home or not, volunteer activity (0), educational level (0), religiosity (0), parental satisfaction (+), hope (+), tolerance (0) | |  | | collective efficacy (0), quality of life (+), perceived economic status (+), relative economic status (0), family size (0), | |
| Finzi-Dottan, Triwitz, & Golubchik, 2011 | CS | ADHD child (71) vs normal' child (80 ') | Israel | 151 | for parents ADHD group: 40.86(6.79);  normal group: 39.86(6.17) | | x | ADHD group: [8.22-13.91];  normal group: [7.80-13.3] | | PSOC | | path analysis | |  | | Emotional Intelligence (+); ADHD (-) | | Social Support (+); | |
| Giallo, Treyvaud, Cooklin, & Wade, 2013 | CS | / | Australia | 851 mothers and 131 fathers | mothers:34.31 (4.64)  fathers:36.49 (4.99)  [19-49] | | 46.2 | [0-4] | | PSOC | | Correlation;  path analysis | | Parent Overall Health (0), stress (-), anxiety (+), depression (-), fatigue (0), marriage quality (0) involvement (+) | | Difficult child temperament (-) | | Perceived support need (-), | |
| Murdock, 2013 | CS | / | America | mothers:49 fathers:33 | mothers:30.45(5.13) [21-45]  fathers:33.85(6.55) [20-52] | | x | 3.73(0.73)  [3-5] | | PSA | | Regression  analysis | | Mothers: general self-efficacy (+), negative affect (0), positive affect (0), hostile or coercive parenting behavior (-), supportive or engaged parenting behavior (0); parental control (0); Fathers: general self-efficacy (+), negative affect (0), positive affect (0), hostile or coercive parenting behavior (0), supportive or engaged parenting behavior (+); parental control (0), marital status (0), | | Mothers: child behavior problems (-), age (0), gender (female: 0) Fathers: child behavior problems (0), age (0), gender (0) | | Mothers: Ethnicity (white 0), family income (0), marital status (single:0), Fathers: ethnicity (0): white or not, family income (0), marital status (0), | |
| Yang, Ke, & Gao, 2020 | CS | / | China | mothers:180 fathers:180 | Mothers:28.59(3.70) Fathers:30.78(3.97) | | 45 | [0.12-0.67] | | PSOC- Efficacy subscale | | regression analysis | | Mothers: parenting satisfaction (+); paternal PSE (+); Fathers: parenting satisfaction (+); maternal PSE (+) | |  | |  | |
| de Haan, Prinzie, & Dekovic, 2009 | L, 6y FU | / | Belgium | T1:594 mothers & 550 fathers  T2: 466 mothers & 426 fathers | T1: mothers:36.1[27.1,52]  fathers:39.0 [27.9,61.1]  T2: mothers [33.1,58];  fathers [33.6,64] | | 50.3 | T1:7.5 [5-10.1];  T2: [11,16.1] | | PSI-competence subscale | | Regression analysis | | gender (mother +); age(0), educational level(0), personality: extraversion(+), agreeableness(+),conscientiousness(0), emotional stability(+), autonomy(+); correlation: overactivity (-) warmth (+) | |  | |  | |
|  |  |  |  |  |  |  |  |  |  |  |  |  | |  |  |  |  |  |  |
| de Haan, Soenens, Deković, & Prinzie, 2013 | L, 8y FU | / | Belgium | mothers:430 fathers:430 | Mothers:36.6 [27-52]  Fathers:41.5 [28-61] | | 49.7 | T1:7.5[6-9]  T2: [12-15]  T3: [14-17] | | PSI-competence subscale | | regression analysis | | Mothers: parental sense of relatedness (+), parental sense of autonomy (+), overreactive discipline (-), warmth discipline(+); age(0); gender (0); Fathers: parental sense of relatedness (+), parental sense of autonomy (+), Parenting discipline (overreactive)(-), Parenting discipline(warmth )(+); age(0); | | Mothers: Age (0), gender (+): girls, aggression (-) Fathers: age (0), gender (+): girls; aggression (-) | |  | |
| Gou, Duerksen, & Woodin, 2019 | L, 2y FU |  | Canada | mothers:74 fathers:72 | Mothers:29.8(5.49); Fathers:32.03(5.51) | | x | t1:0;  T2:1;  T3:2 | | PSOC | | regression  analysis | | gender (0), | |  | | Mothers: partner’s coercive control (0), Fathers: coercive control of partner (-), | |
|  |  |  |  |  |  |  |  |  |  |  |  |  | |  |  |  |  |  |  |
| Gordo et al., 2018 | L, 11m FU | / | Spain | mothers:580 fathers:385 | Mothers:32.77(5.11) Fathers:35.14(5.07) | | x | 0.66(0.12)  [0.42.1.22] | | PSOC-Efficacy subscale | | correlation; path analysis; | | Mothers: Perception of child’s vulnerability (-), depression (-), stress (-); rewards (-), stressors (-), parental competence (+), parental satisfaction(+) Fathers: gender (mothers: +), Perception of child’s vulnerability (+), depression(-), stress(-); rewards(-), stressors(-), satisfaction(+), competence (+) | |  | |  | |
| Knauth, 2000 | L, 8m FU | high risk vs low risk | America | mothers:114 fathers:114 | Mothers:29.3(4.7) Fathers:31.8(7.2) | | x | 0.67 | | PSOC | | Regression  analysis | | Mothers: prenatal family functioning (+), family functioning at T2(0), family functioning at T1(-), parental competence at T1(+), IMP (prenatal/T1/T2) (0) Fathers: prenatal family functioning (0), family functioning at T1(0), family functioning at T2(0), parental competence at T1(+), IMP (prenatal/T1/T2)(0), | |  | |  | |
| Notes: Abbreviations: SD=Standard Definition=; PSOC=Parenting Sense of Competence; PSE=Parenting self-efficacy; PSI=Parenting Stress Index; PSA=Parenting Self-Agency measurement; MBS=Mother and Baby Scale; SPPR=Self-perception of Parent Role questionnaire; CS=cross-sectional; FU=follow-up; L=L; SES=socioeconomic status; y=year; m=month, w=week; X: not reported; /: not applicable; ^†^ age of children has been converted into years, if months or weeks were reported; ^‡^ + or - : statistically non- significant positive or negative association; 0: no association . | | | | | | | | | | | | | | | | | | | |

| **Supplementary Table 2. Results of the quality assessment of the studies included in the systematic review (N=30)** | | | | | | | | | | | | | | |  |
| --- | --- | --- | --- | --- | --- | --- | --- | --- | --- | --- | --- | --- | --- | --- | --- |
| Author and year | 1. Research question | 2. Study design | 3. Subject and variable selection | 4. Subject characteristics | 8. Exposures and outcome | 9. Sample size | 10. Analytic methods | 11. Estimate of variance | 12. Confounding | 13. Results in sufficient detail | 14. Conclusions support results | sums | weights^†^ | Rank # | |
| Baker et al., 2013 | 2 | 2 | 2 | 1 | 2 | 1 | 1 | 0 | 1 | 2 | 2 | 16 | 72.7 | good | |
| Cooklin et al., 2012 | 2 | 1 | 1 | 2 | 2 | 2 | 2 | 1 | 2 | 2 | 2 | 19 | 86.4 | excellent | |
| Cutrona & Troutman, 1986 | 2 | 1 | 1 | 1 | 2 | 1 | 2 | 0 | 1 | 2 | 2 | 15 | 68.2 | adequate | |
| Davidson Arad et al., 2018 | 2 | 1 | 1 | 1 | 2 | 1 | 1 | 1 | 2 | 2 | 2 | 16 | 72.7 | good | |
| de Haan et al., 2009 | 1 | 1 | 2 | 1 | 2 | 2 | 2 | 2 | 1 | 2 | 2 | 18 | 81.8 | excellent | |
| de Haan et al., 2013 | 2 | 2 | 2 | 1 | 2 | 2 | 2 | 2 | 1 | 2 | 2 | 20 | 90.9 | excellent | |
| Dunning & Giallo, 2012 | 2 | 1 | 1 | 2 | 2 | 2 | 2 | 0 | 1 | 2 | 2 | 17 | 77.3 | good | |
| Ercegovac et al., 2013 | 2 | 1 | 2 | 1 | 1 | 2 | 1 | 0 | 2 | 2 | 2 | 16 | 72.7 | good | |
| Finzi-Dottan et al., 2011 | 2 | 1 | 2 | 2 | 2 | 1 | 1 | 2 | 2 | 2 | 2 | 19 | 86.4 | excellent | |
| Giallo et al., 2013 | 2 | 1 | 1 | 2 | 2 | 2 | 2 | 1 | 1 | 2 | 2 | 18 | 81.8 | excellent | |
| Gordo et al., 2018 | 2 | 1 | 1 | 2 | 2 | 2 | 2 | 1 | 1 | 2 | 2 | 18 | 81.8 | excellent | |
| Gou et al., 2019 | 2 | 1 | 1 | 1 | 2 | 1 | 2 | 2 | 1 | 2 | 2 | 17 | 77.3 | excellent | |
| Henney, 2016 | 1 | 2 | 1 | 2 | 1 | 1 | 2 | 0 | 1 | 0 | 1 | 12 | 54.5 | adequate | |
| Hill & Tyson, 2008 | 2 | 2 | 2 | 1 | 2 | 1 | 1 | 2 | 2 | 2 | 2 | 19 | 86.4 | excellent | |
| Holloway et al., 2005 | 1 | 1 | 1 | 1 | 2 | 1 | 2 | 2 | 0 | 2 | 2 | 15 | 68.2 | adequate | |
| Hurwich-Reiss & Watamura, 2019 | 1 | 2 | 2 | 1 | 2 | 2 | 2 | 1 | 1 | 2 | 2 | 18 | 81.8 | excellent | |
| Jover et al., 2014 | 2 | 2 | 1 | 1 | 1 | 1 | 1 | 1 | 1 | 1 | 2 | 14 | 63.6 | adequate | |
| Katkic et al., 2017 | 2 | 1 | 1 | 1 | 1 | 1 | 2 | 1 | 1 | 2 | 2 | 15 | 68.2 | adequate | |
| Knauth, 2000 | 2 | 1 | 1 | 2 | 2 | 1 | 2 | 0 | 1 | 2 | 2 | 16 | 72.7 | good | |
| Kwok & Li, 2015 | 2 | 2 | 1 | 2 | 2 | 2 | 2 | 0 | 2 | 2 | 2 | 19 | 86.4 | excellent | |
| Mazur, 2006 | 1 | 1 | 1 | 1 | 2 | 1 | 2 | 2 | 1 | 2 | 2 | 16 | 72.7 | good | |
| McBride, 1989 | 1 | 1 | 0 | 2 | 1 | 1 | 1 | 0 | 1 | 1 | 2 | 11 | 50.0 | Low | |
| Murdock, 2013 | 1 | 1 | 1 | 2 | 1 | 1 | 2 | 2 | 2 | 2 | 1 | 16 | 72.7 | good | |
| Ogel-Balaban & Altan, 2020 | 2 | 1 | 1 | 1 | 2 | 2 | 2 | 0 | 1 | 2 | 1 | 15 | 68.2 | adequate | |
| Shrooti et al., 2016 | 1 | 1 | 2 | 2 | 2 | 2 | 2 | 2 | 2 | 2 | 1 | 19 | 86.4 | excellent | |
| Studts et al., 2019 | 1 | 1 | 1 | 2 | 2 | 1 | 2 | 2 | 1 | 2 | 2 | 17 | 77.3 | good | |
| Suzuki et al., 2009 | 1 | 1 | 2 | 2 | 1 | 1 | 2 | 2 | 2 | 2 | 2 | 18 | 81.8 | excellent | |
| Teti & Gelfand, 1991 | 1 | 1 | 1 | 1 | 1 | 1 | 2 | 0 | 2 | 2 | 2 | 14 | 63.6 | adequate | |
| Troutman et al., 2012 | 2 | 2 | 2 | 1 | 2 | 1 | 1 | 0 | 1 | 1 | 2 | 15 | 68.0 | adequate | |
| Yang et al., 2020 | 2 | 2 | 2 | 2 | 2 | 2 | 1 | 0 | 0 | 1 | 1 | 15 | 68.2 | adequate | |
| Notes: Each item was scored depending on to what degree the criterion was met: yes = 2 points, partial = 1 point, no = 0.; ^†^ study total sum score divided by the total possible score of 22; # A percentage score of >80%, 70%-80%, 55-69% and <55% was rated as “excellent”, “good”, adequate and “low”, respectively. | | | | | | | | | | | | | | |  |

| **Supplementary Table 3. Mothers, fathers and PSE, results from studies carried among parents with subgroup analysis (n=6)** | | | | |
| --- | --- | --- | --- | --- |
|  |  | **Direction of Associations** ^†^ | | |
|  |  | **Negative (-)** | **Null (0)** | **Positive (+)** |
| **PARENTAL FACTORS** | |  |  |  |
| **Socio-demographic** | |  |  |  |
|  | Gender(female) |  | de Haan et al., 2013 ♀;Gou et al., 2019 ♀;  de Haan et al., 2013 ♂; Gou et al., 2019 ♂; | Gordo et al., 2018 ♀;  Gordo et al., 2018 ♂; |
|  | Age | ***de Haan et al., 2013 ♂*** | ***de Haan et al., 2013 ♀;*** |  |
|  | Ethnicity (native) |  | Murdock, 2013 ♀;  Murdock, 2013 ♂; |  |
| **Parents' Personality/Psychological factors** | | |  |  |
|  | Depression | Gordo et al., 2018 ♀; Gordo et al., 2018 ♂; |  |  |
|  | Parenting stress (higher) | Gordo et al., 2018 ♀; Gordo et al., 2018 ♂; |  |  |
|  | Parenting rewards | Gordo et al., 2018 ♀; Gordo et al., 2018 ♂ |  |  |
|  | Parenting stressors | Gordo et al.,2018 ♀; Gordo et al., 2018 ♂ |  |  |
|  | Parenting self-efficacy (Global level) (higher) |  |  | Murdock, 2013 ♀;  Murdock, 2013 ♂; |
|  | Partners' parenting self-efficacy (higher) |  |  | Yang et al., 2020 ♀;  Yang et al., 2020 ♂ |
|  | Parental competence (higher) |  |  | Knauth, 2000 ♀;  Gordo et al., 2018 ♀; Gordo et al., 2018 ♂; Knauth, 2000 ♂ |
|  | Parental affect |  | Murdock, 2013 ♀; Murdock, 2013 ♂ |  |
|  | Psychological need satisfaction (relatedness) |  |  | de Haan et al., 2013 ♀; de de Haan et al., 2013 ♂; |
|  | Psychological need satisfaction (autonomy) |  |  | de Haan et al., 2013 ♀; de Haan et al., 2013 ♂; |
| **Parental-child interaction** | |  |  |  |
|  | Perception of child’s vulnerability | ***Gordo et al., 2018 ♀*** |  | ***Gordo et al., 2018 ♂*** |
|  | Parenting behavior(control) |  | Murdock, 2013 ♀; Murdock, 2013 ♂; |  |
|  | Parenting behavior (hostile or coercive) | *Murdock, 2013 ♀* | ***Murdock, 2013 ♂*** |  |
|  | Parenting behavior (supportive or engaged) |  | ***Murdock, 2013 ♀*** | ***Murdock, 2013 ♂*** |
|  | Parenting satisfaction (higher) |  |  | Gordo et al., 2018 ♀; Gordo et al., 2018 ♀; Yang et al., 2020 ♀; Yang et al., 2020 ♂ |
|  | Parenting discipline (overreactive ), | de Haan et al., 2013 ♀; de Haan et al., 2013 ♂; |  |  |
|  | Parenting discipline (warmth ); |  |  | de Haan et al., 2013 ♀; de Haan et al., 2013 ♂; |
| **CHILD FACTORS** | |  |  |  |
|  | Age |  | Murdock, 2013 ♀ ; de Haan et al., 2013 ♀ ; Murdock, 2013 ♂; de Haan et al., 2013 ♂; |  |
|  | Gender (girls) |  | Murdock, 2013 ♀; Murdock, 2013 ♂; | de Haan et al., 2013 ♀; de Haan et al., 2013 ♂ |
| **Health** |  |  |  |  |
|  | Behavior problems | ***Murdock, 2013 ♀;*** | ***Murdock, 2013 ♂*** |  |
|  | Aggression (more) | de Haan et al., 2013 ♀; de Haan et al., 2013 ♂ |  |  |
| **SOCIAL CONTEXTUAL FACTORS** | | |  |  |
| **Social Support** | |  |  |  |
|  | Marital status (single) |  | Murdock, 2013 ♀; Murdock, 2013 ♂; |  |
|  | Partner violence (coercive control) | ***Gou et al., 2019 ♂*** | ***Gou et al., 2019 ♀*** |  |
| **Occupation Characteristics** | |  |  |  |
| **Family factors** | |  |  |  |
|  | Household income (higher) |  | Murdock, 2013 ♀; Murdock, 2013 ♂; |  |
|  | Family functioning |  | ***Knauth, 2000 ♂*** | ***Knauth, 2000 ♀;*** |
|  | Perceived Importance for family relationship |  | Knauth, 2000 ♀; Knauth, 2000 ♂ |  |
| Notes: ^†^ summarized data from studies performed in parents with subgroup analysis on mothers and fathers; ♀: Results for mothers; ♂: results for fathers;  Bold and italic indicates different directions of the associations between factors and PSE in mothers and fathers. | | | | |
